# Trauma-related mental health problems among national humanitarian staff: a systematic review of the literature

**DOI:** 10.3402/ejpt.v6.28541

**Published:** 2015-11-19

**Authors:** Hannah Strohmeier, Willem F. Scholte

**Affiliations:** 1 Institute of Tropical Medicine and International Health, Charité – Universitätsmedizin Berlin, Berlin, Germany; 2 Antares Foundation, Amsterdam, The Netherlands; 3 Equator Foundation, Diemen, The Netherlands; 4 Academic Medical Center, University of Amsterdam, The Netherlands

**Keywords:** Mental illness, posttraumatic stress disorder, PTSD, depression, anxiety disorder, substance use disorder, suicide, relief workers, aid workers, humanitarian organization

## Abstract

**Background:**

Working in humanitarian crisis situations is dangerous. National humanitarian staff in particular face the risk of primary and secondary trauma exposure which can lead to mental health problems. Despite this, research on the mental health of national staff is scarce, and a systematic analysis of up-to-date findings has not been undertaken yet.

**Objective:**

This article reviews the available literature on trauma-related mental health problems among national humanitarian staff. It focuses on the prevalence of selected mental health problems in relation to reference groups; sex and/or gender as predictive factors of mental health problems; and the influence of organization types on mental health problems.

**Method:**

Three databases were systematically searched for relevant studies published in the English language in peer-reviewed journals.

**Results:**

Fourteen articles matched the inclusion criteria. Findings suggest that national staff experience mental health problems and the prevalence of posttraumatic stress disorder, depression, and anxiety among this occupation group is mostly similar to or higher than among reference groups. Research on both substance use disorder and suicidal behavior among national staff is particularly scarce. The relation between sex and/or gender and mental health problems among national staff appears to be complex, and organizational staff support seems to be an important determinant for mental health.

**Conclusion:**

All findings call for increased attention from the humanitarian community and further research on the topic.

Working in humanitarian crisis situations is dangerous and staff are increasingly at risk (Connorton, Perry, Hemenway, & Miller, 2012). The latest data collected by “Humanitarian Outcomes” show a new record of violence against civilian aid operations in 2013. A total of 251 separate attacks resulted in 155 dead, 171 seriously wounded, and 134 kidnapped humanitarian workers — an increase of 48% in separate attacks and 66% in the number of victims as compared to 2012 (Stoddard, Harmer, & Ryou, 2014). In addition to the danger of being physically harmed, humanitarian workers face the risk of suffering from primary or secondary traumatic stress as a consequence of these attacks and other traumatic events experienced during employment. Examples of such events are murder of a co-worker, friend, or family member; sniper shots; torture; rape or other forms of gender-based violence (Lopes Cardozo et al., 2005; Shah, Garland, & Katz, 2007).

The vast majority (92%) of humanitarian workers operating in the field are national staff (Stoddard et al., 2014; Taylor et al., 2012). It is, therefore, not surprising that 87% of humanitarian worker victims in 2013 belonged to this occupation group (Stoddard et al., 2014).

Yet, not everyone who experiences one or more traumatic events develops mental health problems. Most people only show temporary symptoms and recuperate readily; it is only a fraction of people who suffer prolonged stress following trauma, which may build the basis for posttraumatic stress disorder (PTSD) or other mental health problems (Davidson & Baum, 1994). If mental health problems manifest, they come with serious implications for the individual as well as his/her social and occupational environment (Brunello et al., 2001). Humanitarian organizations, for instance, increasingly perceive staff stress and compromised mental health as a threat to organizational effectiveness and efficiency (Welton-Mitchell, 2013).

In contrast to research on military personnel, clinicians dealing with patients who have experienced hardship, or other emergency staff, research on trauma-related mental health problems among humanitarian workers is still in its infancy. The topic started to gain attention in the 1990s and since then a limited number of studies have been undertaken. The majority of these studies concentrate on expatriate workers. Trauma-related mental health problems among national staff have received much less attention, although they make up the vast majority of humanitarian workers and are frequently exposed to traumatic events (Ager et al., 2012). An exclusive analysis of studies on trauma-related mental health problems among national staff has not been undertaken yet.

This qualitative, systematic literature review addresses this gap. It explores PTSD as the “signature” disorder victims of traumatic events suffer from. PTSD usually co-occurs with other mental health problems. This review also analyses depression, substance use disorder (SUD), and anxiety disorders, which are recognized as further important posttrauma disorders and the most common comorbid conditions of PTSD (Brady, Killeen, Brewerton, & Lucerini, 2000; Knox, 2008). Given its association with trauma exposure, this review also explores suicidal behavior (Breslau, 2009).

This review centers on three subject areas. Firstly, the prevalence of mental health problems among national staff, specifically in relation to reference groups from similar geographical settings and cultural backgrounds. This approach puts study results into context and offers valuable clues to national staff's risk for mental health problems.

Secondly, sex and/or gender as predictors for mental health problems. Research on demographic risk factors for PTSD in the general population suggests that women are twice as likely as men to develop this condition in their lifetime (Olff, Draijer, Langeland, & Gersons, 2007). Being a woman also increases the risk of developing depression and anxiety (Altemus, 2006), while SUD and sucide are more common among men (WHO 2014, 2015). Information about whether these patterns hold true in the context of national staff is valuable, including for the design of adequate, gender-responsive prevention and response mechanisms.

Thirdly, organization type as determinant of mental health problems. Organization types include, but are not limited to “national non-governmental organization (NGO),” “international NGO,” and “intergovernmental organization.” Organizations of the same type can be diverse but typical disparities between organization types, such as proximity to the epicenter of crises and different staff support strategies, seem to be particularly relevant in predicting mental health problems: front-line responders, for instance from Red Cross/Red Crescent Societies and national NGOs, experience a disproportionally high number of the attacks against civilian aid operations (Stoddard et al., 2014). Cumulative trauma influences the risk of developing PTSD as well as the severity of PTSD and major depression (Kolassa, Illek, Wilker, Karabatsiakis, & Elbert, 2015). Staff support strategies include services such as briefings prior and after assignment, counseling options, and peer helper initiatives. Humanitarian workers find these useful and attribute growing importance to receiving such services (Curling & Simmons, 2010). Evidence suggests that many NGOs lack appropriate support measures for their staff, while United Nations (UN) and related organizations have formalized structures in place that appear to protect from mental health problems (Ager et al., 2012; Ditzler, 2001; Ehrenreich & Elliott, 2004). Insights into whether the organization type has an influence on staff's mental well-being is relevant, including for the development of effective occupational health policies.

A universal definition of national humanitarian staff does not exist (Connorton et al., 2012). This review uses the term “national staff” as nationals of developing countries who provide paid or unpaid humanitarian activities in their homeland through the government or humanitarian organizations.

## Methods

The databases PubMed, PsycINFO, and PILOTS were searched for relevant literature. Regarding the selection of suitable search terms, the established method by Connorton et al. (2012) who undertook a broader literature review on trauma-related mental illness and humanitarian relief workers was applied: given that different terminologies for humanitarian staff are in use, searches were conducted for the terms “relief-workers,” “aid-workers,” and “humanitarian-workers,” in combination (“and”) with the terms “mental health,” “mental illness,” “posttraumatic stress disorder,” “depression,” “anxiety disorder,” “substance use disorder,” and “suicide” anywhere in the text (“in all fields”/“anywhere”).

To be included, studies had to examine at least one of the following areas: PTSD, depressive disorder, anxiety disorder, SUD, or suicidal behavior among national staff. Further inclusion criteria were that the literature is peer-reviewed, published in a journal, and written in the English language. Research was excluded if related to occupation groups such as peacekeepers, military personnel, development aid workers, human rights workers, volunteers not affiliated with a humanitarian organization or governmental humanitarian efforts, and national staff supporting relief efforts in industrialized countries.

The literature search was undertaken in January 2015 without time frame limitations. All search results entered by that time in one or more of the databases that matched the above-mentioned requirements were included in this review. One author of this study conducted the literature search and reviewed titles, abstracts, and where needed, the entire study for suitability. Both authors approved the methodological proceeding and confirmed the final set of articles.

## Results

### Descriptive analysis

After exclusion of unsuitable material for reasons specified above, 14 articles matched the inclusion criteria (Table 1). The numbers in parentheses in this section refer to the article identification number in Table 1.

**Table 1:** Overview of studies included in this review

Author identification number	First author, year	Methodology, method, time frame	Total number of study participants/number or percentage of national staff (sex national staff), subjects and place	Outcome studied (measure)	Prevalence rate national staff (prevalence rate reference group)	Results on sex/gender	Results on organization type	Presented limitations of study	Other relevant information
*Quantitative studies*									
1	Ager, 2012	Quantitative study; cross-sectional; self-administered survey; during service	*N*=376/*N*=376 (64% male, 36% female) National staff from Uganda	PTSD (Los Angeles Symptom Checklist)	26%	Women reported significantly more symptoms of anxiety, depression, PTSD, and emotional exhaustion than men	Working with internat. NGO (compared with working with a UN and related agency) risk factor for depression and anxiety. Proportion of staff of nat. NGOs reporting clinically concerning levels of depression symptoms similar to that of staff of internat. NGOs, but significantly above that of staff of UN and related orgs. Org. type no significant variable to predict PTSD symptoms	Only staff working in organizations with at least 20 national staff participated in survey; cross-sectional design; findings are based upon self-report of symptoms	Increasing exposure to chronic stress associated with increased anxiety symptoms, with most prevalent chronic stressor organizationally related: economic/financial problems, asked to perform duties outside of professional training, lack of recognition for work accomplished by management, tension due to inequality of treatment between expatriate and national staff
				Depression (Hopkins Symptom Checklist)	68% (45 and 67%)* *These rates refer to Ugandan IDPs				
				Anxiety (Hopkins Symptom Checklist)	53%				
				Burnout (Maslach Burnout Index Human Services Survey)	5%				
2	Ehring, 2011	Quantitative study; cross-sectional; self-report questionnaires; during service	*N*=267 / *N*=267 (83.9% male, 16.1% female) National staff from Pakistan	PTSD (Impact of Event Scale-Revised)	42.6%	Women showed significantly higher levels of PTSD, mixed anxiety, and depression on the PADQ, somatic symptoms on the BSI, and burnout. No significant differences were found for depression	n/a (all staff from same organization)	Cross-sectional; no use of structured clinical interviews; questionnaires assessing burnout and social support were problematic from an intercultural perspective	Greater levels of social support associated with lower symptom severities for majority of outcomes measured
				Depression (Pakistan Anxiety and Depression Questionnaire and Bradford Somatic Inventory)	23.3% (PADQ)				
				Anxiety (Pakistan Anxiety and Depression Questionnaire and Bradford Somatic Inventory)	18.8% (PADQ)				
				Burnout (Maslach Burnout Inventory)	7.8%				
3	Eriksson, 2013	Quantitative study; cross-sectional; group-administered survey; during service	*N*=255/*N*=165 (26.4% male, 73.6% female) National staff from Jordan and Iraqi refugee volunteers	PTSD (Los Angeles Symptom Checklist)	19.2%	Women were 4.3 times more likely than men to report clinical levels of anxiety	Organization type as variable was included but not significant	Sample may not be representative; stressors were limited by the predetermined list of stressors; in-depth interviews would have provided more nuanced understanding of well-being of staff; time and financial constrains; cross-sectional design	Important resilience factors for occupational burnout and mental health outcomes: social support and team cohesion
				Depression (Hopkins Symptom Checklist)	48.3%* (31%)** **This rate refers to a random sample of Jordanian women in a primary care setting				
				Anxiety (Hopkins Symptom Checklist)	43.3%* *These rates refer to Jordanian national staff only.				
				Burnout (Maslach Burnout Inventory-Human Services Survey)	5.8%				
4	Hagh-Shenas, 2005	Quantitative study; cross-sectional; questionnaires; 90 days after earthquake	*N*=154/*N*=18 (n/a) Red Crescent workers (=national staff), fire fighters, and student volunteers from Iran	PTSD (Civilian Mississippi Scale, Persian Version ESHEL)	1 out of 18 (1 out of 36 fire fighters; 34 out of 100 students)	n/a	n/a	n/a	Students without affiliation to any org. and formal training had worse psychological effects than Red Cross workers and fire fighters
				Different aspects (General Health Questionnaire)	n/a (means of all subscales greater for students than for Red Crescent workers and fire fighters)				
				Anxiety (Anxiety Sensitivity Index)	n/a (students scored significantly higher than Red Crescent workers and fire fighters)				
5	Lopes Cardozo, 2013	Quantitative study; cross-sectional; group-administered survey; during service	*N*=398/*N*=398 (44.3% male, 55.7% female) National staff from Sri Lanka	PTSD (Harvard Trauma Questionnaire)	19% (7%)*	No statistically significant gender differences were found for the outcomes studied	Staff working for nat. NGOs, the Red Cross, or a UN org. less likely to suffer depression symptoms than staff working for an internat. NGO. Org. type not significantly correlated with PTSD or anxiety	Cross-sectional design; Findings based on self-reported symptoms; screening instruments not specifically validated for Vanni region; findings limited to specific population only	Participants who received less social support more likely to experience probable PTSD than those who received more social support. Probable PTSD significantly more likely among those who experienced greater numbers of conflicts and misunderstandings with co-workers
				Depression (Hopkins Symptom Checklist)	58% (22%)*				
				Anxiety (Hopkins Symptom Checklist)	53% (32%)* *These rates refer to Jaffna district residents				
6	Lopes Cardozo, 2005	Quantitative study; cross-sectional; self-administered survey; during service	*N*=610/*N*=325 (70.5% male, 29.5% female) Expatriates and Kosovar Albanians (=national staff) working in Kosovo	PTSD (Harvard Trauma Questionnaire)	6.15% (17.1%)* (1.1%)**	Women had substantially more depression and worse NSPM than men	n/a	No clinical interviews; timeframe for symptoms of PTSD was only 1 week; qualitative component could have generated important information; response rate was high but non-response bias cannot be ruled out; cross-sectional survey; lack of baseline data and information on mental health prior to recruitment; retrospective studies involving recollection of trauma events may be limited by inaccurate recall; situation in Kosovo was relatively stable and results are not generalizable to relief workers operating in more acute emergencies	Occupational factors may have different relevance for national staff and expatriates: lack of access to org. support services not associated with adverse mental health outcomes among national staff. For national staff, access to regular social support may have been more relevant than org. support
				Depression (Hopkins Symptom Checklist-25)	16.92% (17.19%)**				
				Non-specific psychiatric morbidity (General Health Questionnaire-28)	11.5% (23.9%)**				
				Hazardous alcohol consumption (3 or more standard alcoholic drinks/day)	2.52% (16.2%)** *This rate refers to a population-based sample of Kosovar Albanians **These rates refer to expatriate staff working in Kosovo				
7	Musa, 2008	Quantitative study; cross-sectional; self-report questionnaires; during service	*N*=53/60% (n/a) Expatriates and Sudanese people (=national staff) working in Sudan	Secondary traumatic stress (Professional Quality of Life Questionnaire)	25%*	Women scored higher levels of burnout than men	n/a	n/a	Study recommends that org. managers and directors should create a positive work climate through the provision of training, psychological support offers, and cultural orientation
				Burnout (Worker Burnout Questionnaire)	16%*				
				Nonpsychotic psychiatric disorders (General Health Questionnaire)	50%* *These rates refer to expatriate and national staff. Results showed that national staff suffered significantly more burnout and STS				
8	Shah, 2007	Quantitative study; cross-sectional; survey administered face-to-face in group fashion; 5 months after mass violence in which staff served	*N*=76/*N*=76 (n/a) National staff from India	PTSD (Secondary Traumatic Stress Scale)	8%	n/a (no demographic data collected)	n/a (all staff worked for NGOs)	STSS not validated for this population; possible respondent pool mismatch due to cultural factors; no comparison group and no baseline data available; one NGO received psychotherapy during study and impacts on STSS scoring are unclear; primary traumatic stress as confounder for measuring STS; primary and secondary trauma intermingle and inform each other; survivor guilt may play a part in magnifying STS; DSM-IV valid in some cultures but may be strained in others	Significant differences in PTSD between staff from different NGOs: mean STS score for NGOs recruiting staff with lower socio-economic status significantly higher than that of NGOs with more privileged staff. Distance to epicenter of violence not significant
				Symptoms of secondary traumatic stress (Secondary Traumatic Stress Scale)	100%				
9	Thormar, 2013	Quantitative study; longitudinal; self-report questionnaires; 6, 12, and 18 months post-earthquake	*N*=506/*N*=506 (74% male, 26% female) National staff from Indonesia	PTSD (Impact of Event Scale-Revised)	23% clinical levels*	Gender had a significant effect on depression, and being male was predictive of higher depressive syndromes	n/a (all staff worked for Red Cross)	n/a	Feeling of safety: one of the most important variables in context of PTSD and anxiety: lack of safety measures facilitated development of PTSD and anxiety symptoms
				Anxiety (Hospital Anxiety and Depression Scale)	58% mild cases, 8% moderate levels*				
				Depression (Hospital Anxiety and Depression Scale)	4% moderate levels* *These rates refer to the third assessment at 18 months post-disaster				
10	Wang, Yip, 2013	Quantitative study; retrospective cohort design; self-administered survey; 11 months after earthquake	*N*=70/*N*=70 (70% male, 25.7% female) National staff from China	Suicidal ideation (single item, binary response format)	21.4% (7.1%)* *This rate refers to suicidal ideation in the same national staff asked about suicidal ideation prior to the earthquake	Sex was excluded from the study as it was not significantly related to any variable	n/a	Cross-sectional; possible recall-bias	Study emphasizes the importance of disaster management’s awareness of mental health risks for staff and the relevance of supportive work climate
				Depression (Chinese version of Center for Epidemiological Studies Depression Scale)	n/a				
				Posttraumatic Stress (15-item Chinese version of Impact of Event Scale)	n/a				
				Job burnout (16-item Maslach Burnout Scale)	n/a				
11	Zhen, 2012	Quantitative study; cross-sectional; self-administered questionnaires; within one year after earthquake	*N*=446/*N*=210 (100% female) National staff and disaster-unexposed nurses from China	PTSD (Traumatic Stress Symptom Checklist)	30% (10.2%)*	n/a (100% female participants)	n/a (all participants from same organization)	Potential cohort effects, such as difference in level of education between national staff and unexposed nurses	In exposed staff, psychological distress associated with the difficulty of task performed, for example, proximity to the center of the earthquake resulted in greater distress
				Depression (Traumatic Stress Symptom Checklist)	27.1% (9.7%)* *These rates refer to disaster-unexposed nurses.				
*Qualitative studies*									
12	Bilal, 2007	Qualitative study; case report; after service	*N*=1/*N*=1 (100%m) National staff from Pakistan	Vicarious traumatization (psychiatric examination)	Diagnosed as suffering from depression and secondary trauma	n/a (only one participant)	n/a (only one participant)	n/a	Case report emphasizes importance of recognizing and managing mental health issues of staff at org. level
13	Wang, Chan, 2013	Qualitative study; semi structured interviews; 10 months after earthquake	*N*=25 / *N*=25 (13 males, 12 females) National staff from China	Stress and coping experiences (semi-structured interviews, thematic analysis)	Results categorized by two general themes: perceived sources of stress and coping experiences	n/a	n/a (all government officials)	Interpretation of results is bound to specific sample	Staff experienced strong feeling of fulfilment, purpose, and meaningfulness of relief work, which may help in handling loss and trauma. Atmosphere at work often more positive than at home
*Mixed method study*									
14	Putman, 2009	Study 1: Qualitative study; focus group discussions; during service	Study 1: *N*=26/*N*=26 (35% male, 65% female) National staff from Guatemala	Study 1: Sources of stress related to work and motivators and rewards that help stay engaged in service	Study 1: Most frequently recurring theme was exposure to direct and indirect violence during work; key problems were lack of training, lack of governmental support, lack of emotional support, lack of financial resources to carry out work; key motivators for work were compassion, gods calling, giving back; most rewarding experiences were seeing growth in community, spiritual benefits	Study 1: n/a	Study 1: n/a (all participants from NGOs)	Studies 1 and 2: No random sample; cross-sectional; self-selection to attend traumatic stress management workshops offered prior to survey: possible bias in that those with more distress may have elected to come or found it difficult to attend; self-report questionnaires; work-related support needs, motivators and rewards were not included in survey: these variables are not directly comparable to the survey results due to differences in method and sample; translation of focus groups may have led to losing some nuances of meaning in qualitative data	Study recommends orgs. should protect their staff through specific safety services, specifically in contexts of high exposure to community violence
		Study 2: Quantitative study; cross-sectional; survey; during service	Study 2: *N*=135/*N*=1 35 (36% male, 64% female) National staff from Guatemala and student volunteers	Study 2 PTSD (LA Symptom Checklist)	Study 2: 17%* *This rate refers to national staff and students. The rate is higher than that found among returned international aid workers from five humanitarian agencies and Guatemalan refugees living in camps in Mexico	Study 2: n/a	Study 2: n/a		
				Exposure to community violence (Survey of Children’s Exposure to Community Violence)	Average number of events: 13				
				Burnout (Maslach Burnout Inventory)	n/a				

Eight studies focused exclusively on mental health problems among national staff (1, 2, 5, 8, 9, 10, 12, 13), two examined expatriate and national staff (6, 7) and four centered on national staff and refugee volunteers (3), national staff, firefighters and student volunteers (4), national staff and disaster-unexposed nurses (11), and national staff and student volunteers (14), respectively. Eleven articles used quantitative methods to assess mental health problems (1–11), two applied a qualitative design (12, 13), and one study a mixed-method approach (14).

All quantitative studies used self-report questionnaires to screen for symptoms of mental health problems, whereby the questionnaires differed between studies. Further, assessments took place at different points in time during or after assignment and trauma exposure. Nine studies and the quantitative part of the mixed-method research applied a cross-sectional design (1–8, 14), one study followed a retrospective cohort design (10) and one examined mental health problems at three sequenced post-disaster time points (9). One qualitative study used a case report format (12) and one conducted semi-structured interviews (13). The qualitative part of the mixed-method research relied on focus group discussions (14).

Research took place in China (10, 11, 13), Guatemala (14), India (8), Indonesia (9), Iran (4), Jordan (3), Kosovo (6), Pakistan (2, 12), Sri Lanka (5), Sudan (7), and Uganda (1). All articles were published between 2005 and 2013.

### Mental health outcomes of national staff in relation to reference groups

Ten studies quantified PTSD. Prevalences range from 6.2% among local Kosovar Albanian staff who scored symptoms concordant with PTSD diagnosis (Lopes Cardozo et al., 2005) to 42% clinically relevant PTSD levels in Pakistani relief workers (Ehring, Razik, & Emmelkamp, 2011). The study undertaken by Hagh-Shenas, Goodarzi, Dehbozorgi, and Farashbandi (2005) in Iran concluded that 1 out of 18 Red Crescent staff had a possible diagnosis of PTSD.

While most studies did not present prevalence rates for reference groups, four did. Lopes Cardozo et al. (2013) found that 18.8% of national staff in Sri Lanka's Vanni region scored levels for probable PTSD cases as compared to 7% of the local population living in Jaffna district. Zhen et al. (2012) assessed that 30% of the national Red Cross nurses from China serving in the aftermath of the Wenchuan earthquake met the criteria for PTSD compared to 10.2% of their unexposed peers. Research from Putman et al. (2009) concluded that with 17% presenting symptoms consistent with PTSD diagnosis, national staff operating in Guatemala were more affected by PTSD than Guatemalan refugees living in camps in Mexico. In contrast, 17.1% of the general Kosovar Albanian population experienced PTSD, whereas the prevalence rate among Kosovar Albanian national staff was 6.2% (Lopes Cardozo et al., 2005).

Seven articles assessed the prevalence of depression. Rates range from 4% moderate levels among Indonesian Red Cross volunteers (Thormar et al., 2013) to 68% of Ugandan national staff scoring levels associated with high risk for depression (Ager et al., 2012). Four studies presented depression prevalence of reference groups with their results: 58% of national staff working in Sri Lanka's Vanni region experienced depression symptoms as compared to 22% of Jaffna district residents (Lopes Cardozo et al., 2013). Zhen et al. (2012) reported probable depression rates for disaster-exposed versus unexposed Chinese Red Cross nurses, which are 27.1 and 9.1%, respectively. Assessments among internally displaced people in Uganda identified rates of 45 and 67%. The latter rate was established with the same tool and is similar to the 68% of national staff who scored levels associated with high risk for depression (Ager et al., 2012). Four percent of Indonesian Red Cross workers experienced moderate levels of depression, which is similar or marginally lower than the prevalence among young Southeast Asians (Thormar et al., 2013).

Anxiety prevalences of national staff were presented in five studies: 53% of national staff from Uganda scored levels for high risk for anxiety disorder (Ager et al., 2012), and 52.8% of staff working in Sri Lanka experienced elevated anxiety (Lopes Cardozo et al., 2013). Among Pakistani recovery workers, 18.8% suffered from clinically relevant levels of anxiety (Ehring et al., 2011), 43.3% of Jordanian national staff scored elevated anxiety symptoms (Eriksson et al., 2013), and Indonesian Red Cross workers reported 58% mild and 8% moderate cases of anxiety (Thormar et al., 2013). Thormar et al. (2013) concluded that their findings of 58% mild and 8% moderate cases in relief workers are similar to the prevalence rates among young Southeast Asians. Lopes Cardozo et al. (2013) found that with 53% versus 32%, anxiety prevalence is higher among national staff than among Jaffna district residents.

SUD and suicidal behavior were each analyzed by one study. Only 2.5% of Kosovar Albanian staff were engaged in hazardous alcohol consumption while 16.2% of expatriate workers drank at hazardous levels (Lopes Cardozo et al., 2005). Among Chinese national staff, 21.4% had suicidal ideations within one year post-disaster. This presents a threefold increase as compared before the earthquake, where 7.1% of the same workers had suicidal ideations (Wang, Yip, & Chan, 2013).

### Sex and/or gender-differentiated mental health outcomes

Seven studies focused on sex and/or gender differences of mental health. Most studies investigated “sex” but used the term “gender.” Results are presented using the terminology applied in the studies.

In the study of Ager et al. (2012) on staff in northern Uganda, PTSD, depression, and anxiety symptoms were reported significantly more frequently by women than by men. Lopes Cardozo et al. (2005) who focused on Kosovar Albanian staff found that depression and non-specific psychiatric morbidity were more prevalent among women than among men. Ehring et al. (2011) found no significant differences between women and men's prevalence rates for depression. However, the levels of mixed depression and anxiety, somatic symptoms of anxiety and depression, and PTSD were significantly higher among female Pakistani staff. The results by Eriksson et al. (2013) on national staff in Jordan imply that women were 4.3 times more likely to belong to the group experiencing anxiety of clinically relevant levels.

Thormar et al. (2013) found that in Indonesian Red Cross workers, gender had a significant effect only on depressive symptoms but not on anxiety or PTSD symptoms. Being male was the determinant for greater symptoms of depression.

Wang, Yip, et al. (2013) included sex as an independent variable in their study on national staff in China but dropped it from the binary logistic regression analysis as no significant correlation with any of the other variables was found. The development of probable PTSD, depression, or anxiety was independent from gender based on the results from Lopes Cardozo et al. (2013) on national staff in Sri Lanka.

### Organization type-differentiated mental health outcomes

Three articles presented results on the organization type their study participants worked for and its significance in explaining mental health problems. In their sample of Sri Lankan national staff, Lopes Cardozo et al. (2013) disaggregated data between those employed by the Red Cross, UN organizations, international NGOs, and national NGOs. They found that staff working for an international NGO were more likely to develop depressive symptoms than those employed by any other type of organization. However, organization type was not significantly correlated with anxiety or probable PTSD.

National staff from Uganda who participated in the study of Ager et al. (2012) were employed by either international NGOs, national NGOs, or UN agencies/international organizations. The findings of this study revealed that those working for an international NGO had a higher risk to develop anxiety and depression in comparison to those employed by the UN and related organizations. The type of organization did not influence the development of PTSD symptoms. Further, results presented from analysis undertaken *post hoc* suggest that the fraction of national NGO staff showing clinically relevant depression symptoms was similar to that of international NGO staff and significantly higher than that of staff working for the UN and related organizations.

Eriksson et al. (2013) collected information on the type of organization Jordanian national staff and Iraqi volunteers worked for. Yet, the variable was not significant and the findings were not discussed further.

## Discussion

PTSD was the most commonly assessed outcome in the articles included in this review, followed by depression and anxiety. It stands out that the prevalence for these mental health problems varies greatly between studies. Caution needs to be applied in comparing these results given their generation with different assessment tools (Dominici, Levy, & Louis, 2005). Some of these tools were also not specifically validated for the study population and assessments took place at different points in time during or after assignment and trauma exposure. This demonstrates the lack of standardization in this branch of mental health research and presents a key obstacle in drawing general conclusions. It should be kept in mind that prevalence rates may be inflated by self-reporting questionnaires instead of structured clinical interviews (Koch & Haring, 2008). Since most studies applied a cross-sectional design, causality can hardly be established.

Other key factors explaining the diversity of PTSD, depression, and anxiety rates include that research took place in different geographic locations and cultural contexts. Previous studies found symptoms, interpretation, and response to anxiety disorders and depression to vary widely across the globe and the research points to a relationship between country-specific PTSD rates and value orientation (Burri & Maercker, 2014; Kirmayer, 2001). The study of Burri and Maercker (2014), which found a significant association between value orientation and PTSD across 11 European countries, is only one example illustrating the relevance of cultural context, including when interpreting the results of this review.

It may be important that some staff responded to natural and others to man-made disasters. While both have much in common, former research suggests that psychological consequences of man-made disasters may persist longer than those caused by natural ones (Solomon & Green, 1992). Some of the study participants were exposed to the crisis directly while others were recruited from parts of the country not immediately affected. This aspect relates to a major challenge in research, in that it is barely possible to detangle the amount of hardship experienced by national staff on a personal level as member of a population group directly involved in the crisis from that confronted with as a professional relief worker (Ehring et al., 2011).

Nonetheless, a certain pattern emerges: the results suggest that PTSD, depression, and anxiety exist among national staff. Where studies reported prevalence rates in reference groups, national staff appeared to be similarly or more affected. This observation deserves attention, as organizations have the moral obligation to prevent and address mental health problems among their staff, ensure their well-being, and sustain organizational effectiveness and efficiency (IASC, 2007). The only exception from this trend comes from the study by Lopes Cardozo et al. (2005), who found that PTSD was much more prevalent among the general Kosovar Albanian population than among national staff. The authors explain this with the privileged status of staff who represented a highly educated segment of society with greater access to coping mechanisms (Lopes Cardozo et al., 2005). The findings from Wang, Chan, Shi, and Wang (2013), who conducted interviews with earthquake survivors, who subsequently engaged in relief work, may present an additional explanation. As their statements show, these workers developed optimism and new meaning and purpose in life precisely through their job as relief workers (Wang, Chan, et al., 2013).

Given the strong comorbidity of PTSD and SUD reported in the literature, it is surprising that SUD was only examined by one study (Breslau, 2009; Olff et al., 2007). Research suggests that this comorbidity is even higher in populations like veterans, where 75% of those with lifetime PTSD suffered from lifetime SUD, too (Jacobsen, Southwick, & Kosten, 2001). It seems particularly counterintuitive that the only study focusing on SUD found higher rates of PTSD, anxiety and depression, and lower rates of hazardous alcohol consumption among Kosovar Albanian staff when compared to their expatriate peers (Lopes Cardozo et al., 2005). While the authors do not go into detail, Connorton et al. (2012) suggest the predominance of the Muslim religion in Kosovo as a possible explanation. However, this conflicts with research from Burazeri and Kark (2010) who found that religious affiliation had no effect on the quantity or frequency of alcohol consumption of Albanian Christians and Muslims.

Suicidal behavior was also assessed by one study only. This is surprising given that exposure to traumatic events has been associated with an increased risk of suicidal behavior, specifically among military and veteran populations (Knox, 2008). While general conclusions on the prevalence of suicidal behavior among national staff cannot be drawn based on the limited data available, the worrying finding by Wang, Yip, et al. (2013) of a threefold increase of suicidal ideation among workers post-quake deserves attention and underlines the strong need for further research in this area.

Most studies concluded that female staff reported more symptoms of common mental disorders than men, in line with the literature and research on other population groups in the respective geographic and cultural context (Ager et al., 2012; Eriksson et al., 2013). This reconfirms that female national staff's greater risk of compromised mental health cannot be explained through the characteristics of humanitarian work only (Ager et al., 2012). Musa and Hamid (2008) cite an alternative explanation in the context of burnout, which alludes to women's specific challenge to combine income generation with houshold chores, as well as their greater involvement in relationships and care work. This matches the factors usually cited as contributing to women's greater risk for common mental disorders (WHO, 2015). Of particular importance in the context of PTSD as well as key in explaining why women are disproportionally affected is also their greater exposure to gender-based and sexual violence (Olff et al., 2007). Findings from the qualitative research undertaken by Putman et al. (2009) affirm indeed that direct and indirect exposure to violence poses an issue in work environments. Exposure to violence, specifically the witnessing of abusive situations, was the theme most often mentioned in focus group discussions conducted with mainly female national staff (Putman et al., 2009).

It is surprising that some studies found sex and/or gender to be an insignificant predictor for mental health outcomes. Lopes Cardozo et al. (2013) offer an explanation that refers to the specific resilience of women who chose to work in the humanitarian sector, which may be similar to the resilience of men and greater than that of women participating in mental health surveys from other population groups. Thormar et al. (2013), who found that gender was irrelevant in determining PTSD, make a similar point by adverting to the difference between community samples and samples comprised of military and police personnel. While research on community samples found a greater risk for women to develop PTSD, this was not the case for military and police samples. This suggests that people in general, and women in particular, who chose to engage in emotionally challenging tasks possess a certain level of resilience.

While these explanations seem plausible they also have weaknesses. Firstly, the argument of Thormar et al. (2013) does not explain their second finding, which noted that male workers experienced more depressive symptoms than their female peers. The authors hypothesize that components of Indonesian culture are responsible for this extraordinary finding: the manner of suppressing strong emotions, the expectation on men to solve issues and to be the main breadwinner during a situation of high unemployment, aggravated by disaster, may have put a disproportionally great pressure on men. Secondly, it is debatable to what extent local people really became humanitarian workers by free choice, given the often limited availability of employment options during or after humanitarian crises (UNHCR, 2013). Specifically women frequently face obstacles in making free employment choices due to preexisting cultural norms and gender inequalities (Aolain, 2011).

The ambiguous results indicate that the relation between sex and/or gender and mental health problems among national staff is complex, and specific research on this particular occupation group is needed. Qualitative research would help clarify the characteristics of men and women working in the field of humanitarian aid as well as gender-specific cultural peculiarities and roles. Further, a differentiated use of the concepts “sex” and “gender” is required to avoid confusion (Afifi, 2007).

Very few studies put a specific focus on the organization type and its impact on mental health. In part, this can be explained by the fact that some studies focused on staff from one organization only or from different organizations of the same type. The two studies that discussed this predictor in greater detail are consistent in their finding that the type of organization had no influence on PTSD development, but working for an international NGO was associated with higher levels of depression symptoms (Lopes Cardozo et al., 2013), and depression and anxiety symptoms, respectively (Ager et al., 2012). Lopes Cardozo et al. (2013) refrain from drawing conclusions from this finding but highlight that it deserves consideration. Ager et al. (2012) point to the formalized structures applied by UN and related organizations, hypothesizing that a combination of training and support for managers, stringent human resource guidelines, staff support services and benefits may protect national employees from mental health problems.

While only a couple of studies focused on the organization type, a more detailed analysis of the reviewed literature reveals that specifically components related to staff support did still receive extensive attention. For instance, Hagh-Shenas et al. (2005) did not differentiate between types of organizations but compared experienced and trained staff with volunteers without affiliation to an organization and formal preparation. Their results show that trained national staff were less affected by mental health problems, including PTSD, depression and anxiety, than unprepared volunteers (Hagh-Shenas et al., 2005). This corresponds to the importance of training and preparation that Ager et al. (2012) discuss. Thormar et al. (2013), who focused on Red Cross volunteers, affirm that organizational factors, such as the provision of sound information, good quality equipment, and the feeling of safety were most influential in predicting PTSD and anxiety. The case report from Bilal, Rana, Rahim, and Ali (2007) emphasizes the importance of the awareness and recognition of staff and their vulnerabilities at the organization level. Vice versa, the interviews conducted by Wang, Chan, et al. (2013), with local relief workers from China, illustrate how excessive demands from organizations, such as high workloads, can cause harm due to additional stress and neglect of much-needed self-care among staff.

This article reviewed the available literature on trauma-related mental health problems among national humanitarian staff. The subject appears to be under investigated. Only a restricted number of articles could be included, which is a limitation of this study. Another limitation lies in the compromised comparability of research findings due to a lack of standardization in this branch of mental health research. The impossibility to draw conclusions regarding the causal relationship between trauma exposure and mental health problems is another key limitation of this article. This is due to the methodological nature of the studies reviewed.

## Conclusion

Although only 14 studies fulfilled the inclusion criteria and despite all other limitations, this literature review provides fundamental information for aid workers, organizations, and researchers. Firstly, up to date findings suggest that national staff experience mental health problems and that the prevalence rates of PTSD, depression, and anxiety among this occupation group are mostly similar to or higher than those of reference groups. Secondly, research on both SUD and suicidal behavior among national staff is particularly scarce. Thirdly, ambiguous findings suggest that the relation between sex and/or gender and mental health problems among national staff is complex. Fourthly, the association between organization type and mental health problems is rarely studied but organizational staff support appears to be an important determinant.

These findings call for increased attention of the humanitarian community and further research on the topic. Longitudinal studies are urgently needed to validate the causality of relationships. A more harmonized approach towards research on mental health problems, as might be defined by the Inter-Agency Standing Committee reference group, would be desirable to allow for easier comparison of results and to facilitate the formulation of general conclusions and recommendations. Finally, more mixed method and qualitative research would help to answer open questions and investigate cultural patterns and specifics in greater detail.
